# Alcohol use in adolescence as a risk factor for overdose in the 1986 Northern Finland Birth Cohort Study

**DOI:** 10.1093/eurpub/ckac099

**Published:** 2022-08-16

**Authors:** Maarit K Koivisto, Jouko Miettunen, Jonna Levola, Antti Mustonen, Anni-Emilia Alakokkare, Caroline L Salom, Solja Niemelä

**Affiliations:** Department of Psychiatry, University of Turku, Turku, Finland; Emergency services, TYKS Acute, Turku University Hospital, Turku, Finland; Center for Life Course Health Research, University of Oulu, Oulu, Finland; Medical Research Center Oulu, Oulu University Hospital and University of Oulu, Oulu, Finland; Department of Psychiatry, University of Helsinki and Helsinki University Hospital, Helsinki, Finland; Center for Life Course Health Research, University of Oulu, Oulu, Finland; Faculty of Medicine and Health Technology, University Consortium of Seinäjoki, Tampere University, Tampere, Finland; Department of Psychiatry, Seinäjoki Central Hospital, Seinäjoki, Finland; Department of Psychiatry, University of Turku, Turku, Finland; Center for Life Course Health Research, University of Oulu, Oulu, Finland; Institute for Social Science Research, The University of Queensland, Brisbane, Australia; Department of Psychiatry, University of Turku, Turku, Finland; Addiction Psychiatry Unit, Department of Psychiatry, Hospital District of South-West Finland, Turku, Finland

## Abstract

**Background:**

Overdoses and poisonings are among the most common causes of death in young adults. Adolescent problem drinking has been associated with psychiatric morbidity in young adulthood as well as with elevated risk for suicide attempts. There is limited knowledge on adolescent alcohol use as a risk factor for alcohol and/or drug overdoses in later life.

**Methods:**

Here, data from The Northern Finland Birth Cohort 1986 study with a follow-up from adolescence to early adulthood were used to assess the associations between adolescent alcohol use and subsequent alcohol or drug overdose. Three predictors were used: age of first intoxication, self-reported alcohol tolerance and frequency of alcohol intoxication in adolescence. ICD-10-coded overdose diagnoses were obtained from nationwide registers. Use of illicit drugs or misuse of medication, Youth Self Report total score, family structure and mother’s education in adolescence were used as covariates.

**Results:**

In multivariate analyses, early age of first alcohol intoxication [hazard ratios (HR) 4.5, 95% confidence intervals (CI) 2.2–9.2, *P* < 0.001], high alcohol tolerance (HR 3.1, 95% CI 1.6–6.0, *P* = 0.001) and frequent alcohol intoxication (HR 1.9, 95% CI 1.0–3.4, *P* = 0.035) all associated with the risk of overdoses. Early age of first intoxication (HR 5.2, 95% CI 1.9–14.7, *P* = 0.002) and high alcohol tolerance (HR 4.4, 95% CI 1.7–11.5, *P* = 0.002) also associated with intentional overdoses.

**Conclusions:**

Alcohol use in adolescence associated prospectively with increased risk of overdose in later life. Early age of first intoxication, high alcohol tolerance and frequent alcohol intoxication are all predictors of overdoses.

## Introduction

Globally, intentional drug overdoses are the most common method of self-harm and/or suicide, the latter is the second most common cause of death among young people.^1^^,^^2^ Overdoses and poisonings are among the most common reasons for substance-use-related emergency room visits.^3^ Fatal overdoses, intentional and unintentional, are common especially among young men of the lowest socioeconomic groups.^4^

Adolescent problem drinking has been associated with mental health problems and psychiatric morbidity in young adulthood^5–7^ as well as with elevated risk for suicide attempts.^8–10^ In previous studies, young age of first intoxication^11–13^ and adolescent binge drinking^14^^,^^15^ have been associated with higher risk of substance-use disorders (SUD) in later life. High alcohol tolerance is considered as one of the first symptoms of alcohol dependence^16^^,^^17^ and is also linked to higher psychiatric morbidity.^7^ Early age of first intoxication, high alcohol tolerance and frequent intoxications in adolescence have been associated with premature mortality in Finland.^18^^,^^19^

There is limited knowledge about the relationship between adolescent substance-use behaviour and non-fatal overdoses in young adulthood. Previous studies concerning risk factors for drug overdose have focussed on solely adult populations^20–23^ or cohorts of adolescents reporting high-risk substance use, e.g. injecting drugs.^21^^,^^24^ Those studies have suggested that specific substance-use characteristics and behaviours are significant risk factors for overdose in young people.^25^ Furthermore, high alcohol consumption has been shown to be independently associated with non-fatal overdoses among young people who inject drugs.^24^ Nevertheless, to date adolescent alcohol use as a risk factor for intentional and unintentional overdoses and poisonings has not been studied in longitudinal general population studies.

In this study, we used data from the Northern Finland Birth Cohort 1986 (NFBC86)^26^ to investigate the prospective association between self-reported age of first intoxication, alcohol tolerance and frequency of alcohol intoxication in mid-adolescence with register-based overdose or poisoning diagnosis by the age of 32–33 years. Alcohol tolerance was determined by self-reported number of drinks required to experience intoxication. We studied the predictive associations of the adolescent reported age of first intoxication, self-reported number of drinks required to achieve inebriation and frequency of alcohol intoxication over the past 30 days with survival from overdoses up to early adulthood. To study the robustness of these relationships, we adjusted for a range of known confounders, such as illicit drug use and family structure.

## Methods

NFBC1986 is an ongoing follow-up study of 99% of all births, including all live-born children (*n* = 9432) with an expected birth between 1 July 1985 and 30 June 1986, from the two northernmost provinces in Finland.^26^ The data on alcohol use were collected in two parts: first by a postal questionnaire,^27^ then by a field study where the participants completed a Supplementary questionnaire including questions on their alcohol use.^28^ Participants were included in the study if they signed the informed consent form. Although there is limited scientific evidence suggesting that a non-fatal overdose is a risk factor for recurring overdose,^29^^,^^30^ we limited the study to individuals with no history of overdosing prior to the age of 15–16 years (*n* = 9402).

**Figure 1:**
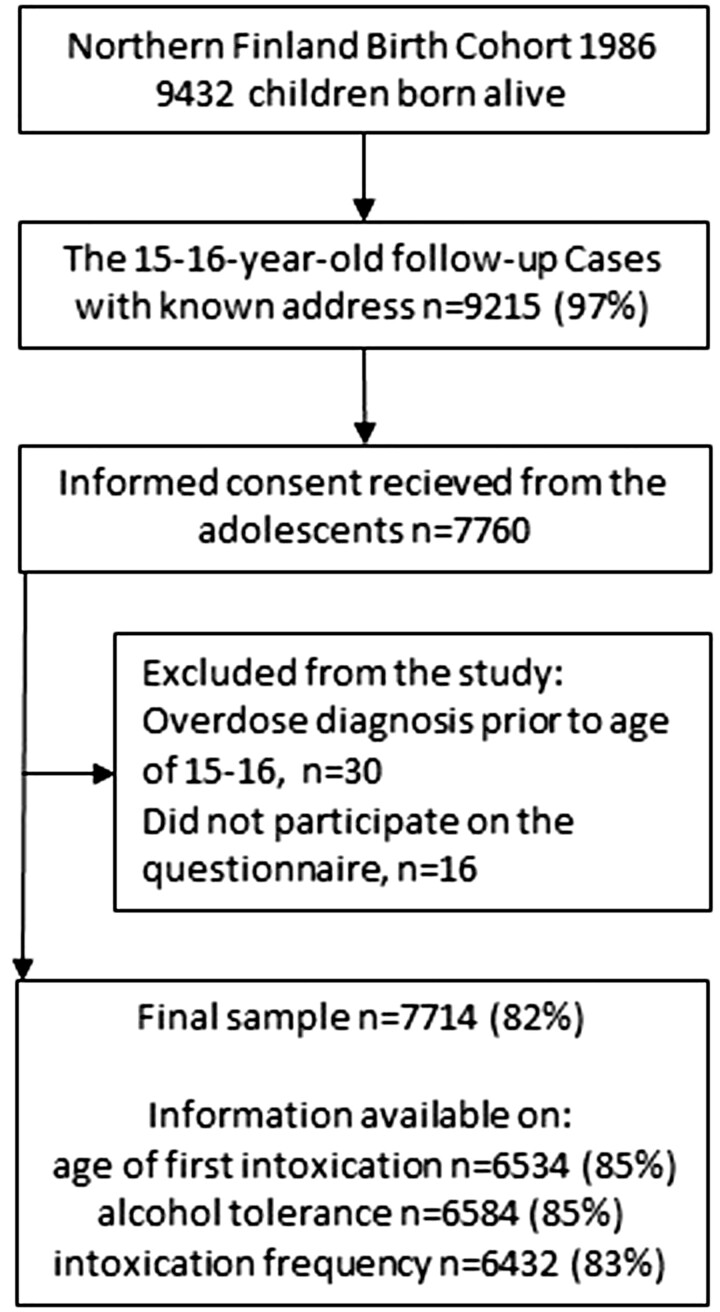
Flowchart of the study

The final sample included 7714 participants with information available on the age of first intoxication *n* = 6534 participants, alcohol tolerance *n* = 6584 participants and frequency of alcohol intoxication *n* = 6432 participants (figure 1). Information on overdose diagnoses was collected cumulatively from nationwide registers from the participant age 15–16 years until 31 December 2018 (age 32–33 years). The study was approved by the Ethics Committee of the Northern Ostrobothnia Hospital District in Finland (15 January 2018, EETTMK 108/2017).^31^

The age of first intoxication was assessed with the question ‘At what age did you get drunk for the first time?’ with options ‘(1) Never, (2) 11 years or younger, (3) 12 years, (4) 13 years, (5) 14 years, (6) 15 years, (7) 16 years’.^27^ Responses were pooled into one four-classed variable: (i) no intoxication, (ii) 12 years or younger, (iii) 13–14 years and (iv) 15–16 years. In this cohort, 7% of the study population reported their age of first intoxication as 12 years or younger.

Alcohol tolerance was assessed with the question: ‘How many drinks do you need to get drunk?’ with options: ‘(1) I have never drunk alcohol, (2) I have never been drunk, (3) 1–2 drinks, (4) 3 drinks, (5) 4 drinks, (6) 5 drinks, (7) 6 drinks, (8) 7–8 drinks and (9) 9 drinks or more’.^28^ One drink was defined as a beverage containing 12 g of pure alcohol.^32^ The participants were given an illustrated example of what constitutes ‘one drink’. Responses were pooled into three predictive variables: (i) no alcohol use or intoxication; (ii) low tolerance group (i.e. below the specified cut off); and (iii) high tolerance group (i.e. equal to or above the specified cut off number of drinks needed to become intoxicated). In many studies, heavy drinking has been defined as more than 4–6 drinks for women and 5–7 drinks for men.^33^ In this study, we set the cut off to seven drinks for females and nine drinks for males. This cut off has been used in previous studies on the same population,^7^^,^^19^ and limits the group with high alcohol tolerance to 11% of the study population, while 4–6 drinks for females and 5–7 drinks for males includes almost 50% of the study population in the high tolerance group.

Frequency of alcohol intoxication was assessed with the question: ‘How many times have you been drunk during the past 30 days?’^28^ Response options were: ‘(1) Never, (2) 1–2 times, (3) 3–5 times, (4) 6–9 times, (5) 10–19 times, (6) 20–39 times or (7) 40 times or more’, and this was categorized as a three-class variable: (i) never, (ii) 1–2 times and (iii) three or more times. This is parallel to the classes used with alcohol tolerance, with ∼10% of the cohort members reporting being drunk three or more times during the last 30 days.

Data on diagnosed overdoses (ICD-10) until the age of 32–33 years were obtained from linkage to nationwide registers: The Care Register for Health Care 2001–2018 of the National Institute for Health and Welfare and The Register of Primary Health Care visits 2011–2018. The Care Register contains information on patients discharged from inpatient care and specialized outpatient care. The Register of Primary Health Care visits includes all outpatient primary health care administered in Finland.

Subjects were included in the overdose group if they had received any of ICD-10-coded diagnoses T36–T50 ‘Poisoning by drugs, medicaments and biological substances or Toxic effects of alcohol’ T51 and Y91. To ensure that no cases were lost in this stage, we also included codes X41, X45, X44, X49X61, X62, X69, X84, X85, X90 and Y57 that are external causes of morbidity and mortality referring to poisonings.^34^ The overdose was classified as intentional if ICD-10-codes X60–X69 ‘Intentional self-poisoning’ or X84 ‘Intentional self-harm by unspecified means’ were used to classify the external cause of overdose or poisoning.^34^ The overdose was classified as unintentional if any of the following ICD-10-codes were used X40–X49 ‘Accidental poisoning’, X85 or X90 ‘Assault by drugs, medicaments, biological substances or unspecified chemical or noxious substance’, Y19 ‘Poisoning by and exposure to other and unspecified chemicals and noxious substances, undetermined intent’ or Y40–Y59 ‘Drugs, medicaments and biological substances causing adverse effects in therapeutic use’.^34^ The diagnosis was taken into account whether or not it was the primary diagnosis of the visit. Individual cohort members may have been included in multiple outcome groups.

Data on lifetime substance use by age 15–16 years were collected using a questionnaire during the field study. The participants were asked about cannabis use (‘Have you used marihuana or hashish?’) and other illicit drug use (‘Have you used ecstasy, heroin, cocaine, amphetamine, LSD or other similar intoxicating drugs?’). The use of inhalant drugs was assessed by the question ‘Have you ever tried sniffing thinner, glue, etc. for intoxication?’ The misuse of medication was assessed by two questions ‘Have you ever tried or used medicines (sedatives, sleeping pills, or pain killers) for intoxication?’ and ‘Have you ever used alcohol and pills together?’ The participant was included in the positive group for each substance type if he/she answered ‘yes’ to any relevant question.

Adolescent behavioural and emotional problems were assessed in the field study at the age of 15–16 years using the Youth Self report (YSR) questionnaire,^35^ with the total score used as a continuous variable. Data on family background were gathered when the cohort members were aged 15–16. The family structure was classified as (i) both parents living with the subject all the time and (ii) all other families. Parental education level and alcohol use were assessed at age 15–16 by a questionnaire completed by mothers and fathers individually. Education level of each parent was divided into two groups: (i) schooling for at least 12 years and (ii) schooling for <12 years. Parental alcohol use was divided into three variables: (i) no alcohol use, (ii) less than once a week and (iii) once a week or more often.

The associations between overdoses and categorical variables describing substance use or background variables were studied using Pearson’s Chi-Square test or Fischer’s exact test and continuous variables with Mann–Whitney U test. Only those variables that statistically significantly associated (*P* < 0.05) with overdoses at univariate analyses were included in further models. The data were then analyzed using Cox regression analysis with hazard ratios (HR) and 95% confidence intervals (95% CI). Times at emigration or death (*n* = 292) were used as censoring points in survival analyses. The reference group consisted of abstinent adolescents who reported no experiences with alcohol. The probability of surviving without overdose in the study groups was determined with adjusted Cox regression survival analyses from age 15–16 years to 32–33 years. Three separate models were built with which the multivariate analyses were performed. First, for Models (1a) the age of first intoxication, (1b) alcohol tolerance and (1c) alcohol intoxication frequency, family structure and mother’s education level were included as independent variables. Models (2a) the age of first intoxication, (2b) alcohol tolerance and (2c) alcohol intoxication frequency were also adjusted for use of drugs (cannabis, inhalant drugs, other illicit drugs or misuse of medication). Models (3a) the age of first intoxication, (3b) alcohol tolerance and (3c) alcohol intoxication frequency were further adjusted for YSR-total score. The statistical analyses were performed using SPSS statistical software (IBM SPSS Statistics, version 24; IBM Co., Armonk, New York, USA).

Attrition analyses regarding data collection at age 15–16 years have been presented previously. Fewer males than females participated (64% vs. 71%; χ^2^ test, *P* < 0.001), as did participants living in urban areas (66% vs. 71%, *P* < 0.001) and adolescents with parental psychiatric disorder (58% vs. 69%, *P* < 0.001).^36^ The final outcomes were based on nationwide registers where there were no missing data. Emigration and deaths during follow-up were scarce.

## Results

Information on self-reported alcohol use at age 15–16 years, overdose diagnoses and potential confounders are presented in table 1. By the age of 32–33 years, there were 183 recorded overdose diagnoses, of which 83 were classified as intentional and 57 as unintentional. Half (50%, *n* = 93) of those diagnosed with overdose were male.

**Table 1:** Sociodemographic characteristics and substance use at age 15–16 and register-based overdoses (intentional and unintentional) by the age of 32–33 years

	All overdoses *n* = 183			Intentional overdoses *n* = 83			Unintentional overdoses *n* = 57		
	*N*	%	*P*-value	*n*	%	*P*-value	*n*	%	*P*-value
Gender									
Male	93	51	0.80	50	60	0.058	27	47	0.74
Female	90	49		33	40		30	53	
Family structure									
Two parents	92	58	<0.001	42	57	<0.001	28	58	<0.001
Other	66	42		32	43		20	42	
Mother’s education									
<12 years	115	78	0.005	59	87	0.001	32	73	0.48
12 years or more	32	22		9	13		12	27	
Father’s education									
<12 years	115	81	0.99	51	81	0.99	34	79	0.72
12 years or more	27	19		12	19		9	21	
Mother’s alcohol use									
No	29	19	0.39	15	21	0.20	7	15	0.46
Less than weekly	100	65		48	69		31	66	
At least weekly	24	16		7	10		9	19	
Father’s alcohol use									
No	25	17	0.75	13	19	0.76	9	21	0.72
Less than weekly	69	48		28	42		17	40	
At least weekly	51	35		26	39		17	40	
Age of first intoxication									
Never	20	14	<0.001	8	13	<0.001	7	14	0.001
15–16 years	19	14		4	7		5	10	
13–14 years	66	48		28	47		29	58	
12 years or younger	33	24		20	33		9	18	
Number of drinks needed to feel intoxicated^a^									
No intoxication	21	15	<0.001	8	13	<0.001	7	14	<0.001
Below cut off	80	58		33	54		29	58	
Over cut off	38	27		20	33		14	28	
Frequency of alcohol intoxicationduring last 30 days									
0	56	41	<0.001	24	40	<0.001	15	31	<0.001
1–2	49	36		21	35		19	39	
3 or more	32	23		15	25		15	31	
Cannabis									
No	114	83	<0.001	54	90	0.15	38	76	<0.001
Yes	24	17		6	10		12	24	
Inhalant drugs									
No	122	88	<0.001	53	88	0.003	41	82	<0.001
Yes	16	12		7	12		9	18	
Other drugs									
No	133	96	0.001	58	97	0.040	47	94	0.002
Yes	5	4		2	3		3	6	
Misuse of medication									
No	110	80	<0.001	46	77	<0.001	36	72	<0.001
Yes	28	20		14	23		14	28	
YSR-total^b^	Mean/median	Range	*P*-value	Mean/median	Range	*P*-value	Mean/median	Range	*P*-value
	35/31	0–98	<0.001	39/35	0–98	<0.001	33/30	8–81	0.005

Categorical variables tested with χ^2^-test or Fischer’s exact test and continuous variables with Mann–Whitney U test.

a Participants in low alcohol tolerance group needed 6/8 drinks or less for females/males and in high alcohol tolerance group 7/9 drinks or more for females/males to become intoxicated.

b Information for Youth Self Report (YSR) are reported as continuous variables.

In bivariate associations age of first intoxication, number of drinks needed to feel intoxicated and alcohol intoxication frequency each were associated with intentional, unintentional and any overdose outcomes (*P* < 0.001; table 1), as were use of cannabis and all other substance types. Family structure associated significantly (*P* < 0.001) with each outcome. Paternal education level was not associated with overdoses. However, maternal education level was associated significantly with any overdoses (*P* = 0.005) and intentional overdoses (*P* = 0.001). Mean and median YSR-total score were significantly higher among those who were diagnosed with any overdose (*P* ≤ 0.005). Neither gender nor parental alcohol use was not associated significantly with any of the overdose outcomes.

Three different Cox regression models were constructed. In Model 1, where we adjusted for family structure and mother’s education level, young age of first intoxication (12 years or younger), high alcohol tolerance and frequent alcohol intoxication past 30 days were statistically significantly associated with higher risk for all the overdose outcomes (Supplementary table S1). In Model 2, use of drugs (cannabis, inhalant drugs, other illicit drugs or misuse of medication) was added to the previous model. The associations were similar with those in Model 1 and remained significant for intentional overdoses but attenuated to statistically non-significant for unintentional overdoses. For the risk of intentional overdoses, association with young age of first intoxication and high alcohol tolerance remained significant but alcohol intoxication frequency did not (Supplementary table S1).

Finally, in Model 3, the YSR-total score was added to the previous models (table 2). After this adjustment the age of first intoxication of 12 years or younger remained a statistically significant risk factor for overdoses (HR 4.5, 95% CI 2.2–9.2, *P* < 0.001, table 2) and intentional overdoses (HR 5.2, 95% CI 1.9–14.7, *P* = 0.002, table 2). The risk for overdoses was also elevated in the group where first intoxication occurred at 13–14 years (HR 2.1, 95% CI 1.2–3.8, *P* = 0.014, table 2). High alcohol tolerance was associated significantly with elevated risk for overdoses (HR 3.1, 95% CI 1.6–6.0, *P* = 0.001, table 2) and intentional overdoses (HR 4.4, 95% CI 1.7–11.5, *P* = 0.002, table 2).

**Table 2:** Alcohol use at the age of 15–16 and overdoses requiring medical attention by age 32–33 years. This model in Cox regression multivariate analyses was adjusted with family structure, mother’s education, lifetime use of drugs (cannabis, inhalants, other illegal drugs or misuse of medication) and YSR^a^-total score

		All overdoses			Intentional overdoses			Unintentional overdoses		
		Events	HR	95% CI	Events	HR	95% CI	Events	HR	95% CI
	vs. ≤12 years	101	4.5	2.2–9.2	41	5.2	1.9–14.7	34	1.6	0.4–6.2
Age of first intoxication	vs. 13–14 years		2.1	1.2–3.8		1.9	0.8–4.6		1.8	0.7–4.6
No intoxication	vs. 15–16 years		1.4	0.7–3.1		0.8	0.2–3.0		0.9	0.2–3.4
Family structure	vs. other		1.9	1.3–2.9		1.9	1.0–3.6		2.0	1.0–4.0
Two parents										
Mother’s education	vs. <12 years		1.5	0.9–2.3		2.2	1.0–5.0		1.5	0.7–3.3
≥12 years										
Drugs	vs. yes		1.3	0.8–2.1		0.7	0.3–1.5		2.2	1.0–4.9
No										
YSR-total^a^			1.02	1.01–1.03		1.03	1.01–1.04		1.02	1.00–1.04
Number of drinks needed to feel intoxicated	vs. over cut off	101	3.1	1.6–6.0	41	4.4	1.7–11.5	34	1.7	0.5–5.6
No intoxication	vs. below cut off		1.6	0.9–2.9		1.3	0.5–3.2		1.4	0.5–3.5
Family structure	vs. other		2.0	1.3–3.0		2.0	1.1–3.8		2.0	1.0–4.0
Two parents										
Mother’s education	vs. <12 years		1.5	0.9–2.3		2.2	1.0–4.9		1.5	0.7–3.3
≥12 years										
Drugs	vs. yes		1.4	0.9–2.3		0.7	0.3–1.5		2.3	1.1–5.2
No										
YSR-total^a^			1.02	1.01–1.03		1.03	1.01–1.05		1.02	1.00–1.04
Frequency of intoxication during last 30 days	vs. ≥3 times	100	1.9	1.0–3.4	41	2.2	0.9–5.3	33	2.46	0.9–6.6
0 times	vs. 1–2 times		1.5	1.0–2.3		1.4	0.7–2.8		1.7	0.7–3.7
Family structure	vs. other		2.1	1.4–3.1		2.1	1.1–4.0		1.8	0.9–3.7
Two parents										
Mother’s education	vs. <12 years		1.5	0.9–2.3		2.3	1.0–5.1		1.4	0.7–3.2
≥12 years										
Drugs	vs. yes		1.5	0.9–2.4		0.8	0.3–1.8		2.1	0.9–4.8
No										
YSR-total^a^			1.02	1.01–1.03		1.03	1.01–1.05		1.02	1.00–1.04

a Youth Self Report.

In Model 3, family structure other than two-parent, maternal education level <12 years and the YSR total score all remained significantly associated with elevated risk for overdoses (table 2) and intentional overdoses (table 2). Use of drugs (cannabis, inhalant drugs, other illegal drugs or misuse of medication) by age 15–16 was the only confounder to remain independently associated with unintentional overdoses (table 2).

**Figure 2:**
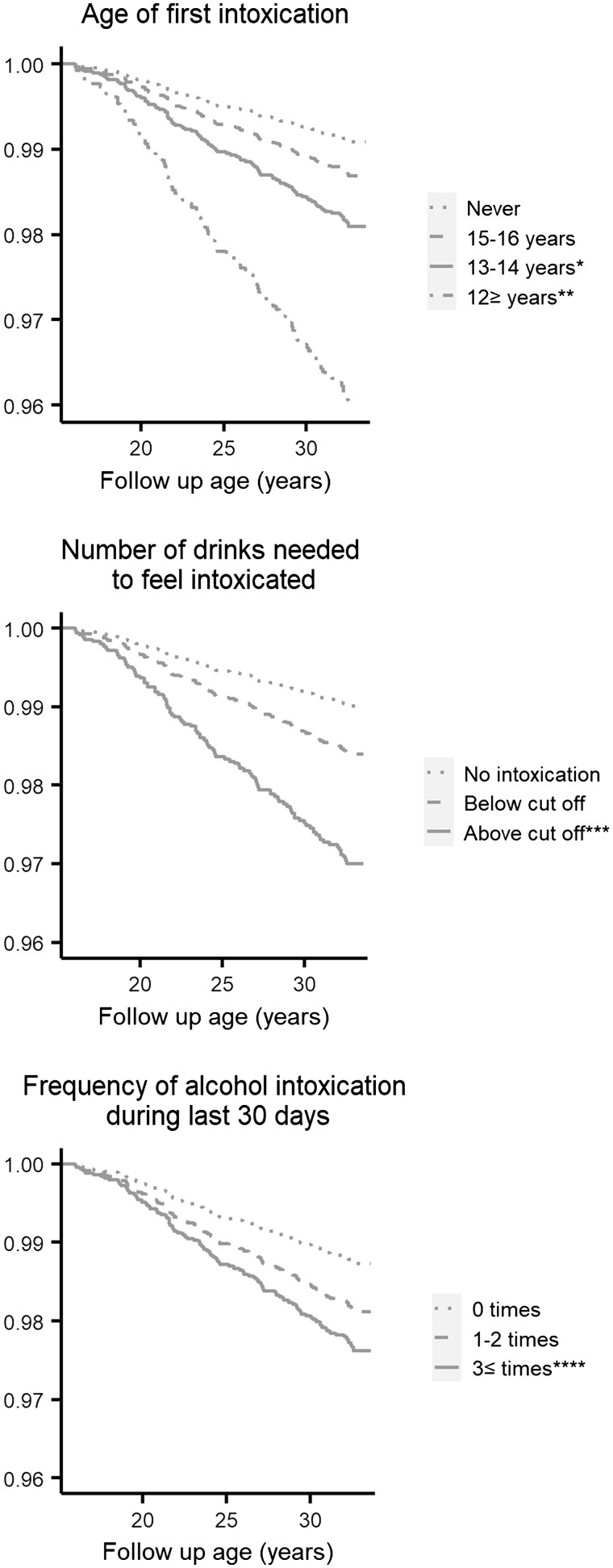
Overdose free survival, intentional overdose free survival and unintentional overdose free survival as functions of the age of first intoxication, number of drinks needed to feel intoxicated and frequency of alcohol intoxication

Survival curves of Models (3a) the age of first intoxication, (3b) alcohol tolerance and (3c) alcohol intoxication frequency are presented in figure 2.

## Discussion

In this large longitudinal birth cohort study, we studied the relationship between self-reported age of first intoxication, alcohol tolerance and alcohol intoxication frequency at age of 15–16 years and the risk of overdoses requiring medical attention by the age of 32–33 years. Here, our findings point out that young age of first intoxication, high alcohol tolerance and frequent alcohol intoxication in adolescence are risk factors for intentional overdoses later in life, independently of behavioural problems, illicit drug use and family background.

In this study, young age of first intoxication was associated independently with overdoses and intentional overdoses. Previous work in other populations has shown early age of first intoxication as a risk factor for alcohol use disorder (AUD)^11^^,^^12^ that in turn increases the risk of suicide attempts.^8–10^ The association between suicide attempts and non-fatal intentional overdoses of medical substances has been established in multiple previous studies.^1^^,^^8^^,^^9^^,^^21^ Age of first intoxication has also been associated with higher risk of psychiatric disorders^13^ and premature mortality.^18^

Self-reported high alcohol tolerance was also associated with overdoses and intentional overdoses. High alcohol tolerance has been associated with higher-dose alcohol consumption.^16^^,^^17^ In a study on a cohort of heavy-drinking young adults, those with high alcohol tolerance experienced less alcohol related adverse events.^16^ This might be encouraging such individuals to persist with high-risk drinking that will eventually lead to adverse events and the development of AUD in later life. This is supported by our finding that high alcohol tolerance is an independent risk factor for the same outcomes as AUD. Frequent intoxication in adolescence has also been shown to associate with elevated risk of AUD^14^^,^^15^ and SUD^7^^,^^15^ in addition to psychiatric comorbidity.^5–7^ AUD and SUD are well documented risk factors for overdoses among both adults^20–23^ and adolescents.^24^^,^^25^ This is in line with our finding that frequent intoxication increases the risk of overdoses.

In this study, lifetime use of cannabis and other drugs were the only independent risk factors for unintentional overdoses. There is some previous evidence to support this finding.^22^^,^^24^^,^^25^ Here, cannabis use was not associated with subsequent intentional overdoses during follow-up. This is in line with the results of a previous birth cohort study on the same population as this study that found a robust association between adolescent (15–16 years) cannabis use and subsequent self-harm requiring medical attention by the age 32–33 but there was not such association found between adolescent cannabis use and death by suicide.^37^ In this study, family structure of other than two parents and mother’s low education level were independent risk factors for overdoses and intentional overdoses, which was expected due the previously demonstrated associations with these family background factors and AUD.^4–7^^,^^11^^,^^12^^,^^14–17^

Interestingly, in this study gender did not associate with any of the outcomes. This is contrary to previous findings suggesting female gender to be an independent risk factor for non-fatal overdoses especially by medications other than opioids.^22^^,^^23^ This finding may be explained by exclusion of those with overdose prior to the age 15–16, lesser participation of males or may relate to the greater representation of males in the heavy-drinking groups.

This study has certain limitations. The information on alcohol use is self-reported and no objective measurements of blood alcohol level were done, but other studies have commented favourably on the reliability of self-reporting of alcohol consumption by adolescents.^38^ The information on the frequency of intoxication was retrospectively estimated by the participants. Also, the first age of use of other substances than alcohol was not asked in the questionnaire and was thus unknown. The data in national registers are generally reliable but under-recording of subsidiary diagnoses is a known limitation for register data.^39^ A particular problem with overdoses is that the substance causing the symptoms may remain unrecognized by the clinician and thus not be included in the ICD-10 diagnoses of the health care visit. Substance overdose is a challenging diagnosis for the clinicians in emergency departments due the complexity of the clinical presentation of the condition.^40^ Variety of different entities were included in the unintended overdoses. It remains unclear how explicitly the possible intention behind the diagnosed overdose was determined by the clinicians.

The strengths of this study are its longitudinal prospective design with considerable follow-up time, the large sample size in a genetically homogenous general population cohort and its use of linked data from multiple registers. In this study, the relationships were able to be adjusted for range of confounders and multiple alcohol use markers allowed us to identify the different facets of risky alcohol use that contribute to risk of overdose.

## Conclusions

High-risk alcohol use in adolescence associates predictively with the risk of overdose in later life. Early onset of drinking, high alcohol tolerance and frequent intoxication are all predictors of overdoses and especially intentional overdoses, which are frequently linked with suicidal behaviour and suicide attempts. In order to prevent overdoses among young adults, early detection and intervention in high-risk alcohol consumption and use of drugs in childhood and adolescence are highly recommended. Overdose prevention is likely to very substantially reduce substance-related mortality.

## Supplementary data

Supplementary data are available at *EURPUB* online.

## Supplementary Material

ckac099_Supplementary_Data
