# Supplementary material for: Alcohol use in adolescence as a risk factor for overdose in the 1986 Northern Finland Birth Cohort Study
Source: Eur J Public Health. 2022 Aug 16;32(5):753–9. doi: 10.1093/eurpub/ckac099 (PMC9527972; doi:10.1093/eurpub/ckac099)
Supplement: ckac099_Supplementary_Data [file ckac099_supplementary_data.docx]

**Supplementary table 1** Alcohol drinking at the age of 15-16 years predicts overdose. In multivariate analyses 3 different models were used. Model 1: adjusted with family structure and mother’s education, Model 2: adjusted with family structure, mother’s education, and use of cannabis or other illicit or prescription drugs Model 3: adjusted with family structure, mother’s education, and use of drugs (cannabis, inhalant drugs, other illicit or misuse of medication) and YSR^1^-total score.

|  | | **All overdoses** | | | **Intentional overdoses** | | | **Unintentional overdoses** | | |
| --- | --- | --- | --- | --- | --- | --- | --- | --- | --- | --- |
|  |  | n | HR | CI95% | n | HR | CI95% | n | HR | CI95% |
| 1. | **Age of first intoxication**  *No intoxication vs.≤12 yrs.* | 25 | 6.3 | 3.4-11.8 | 14 | 8.2 | 3.3-20.8 | 6 | 4.2 | 1.3-13.2 |
|  | **Number of drinks needed to feel intoxicated**  *No intoxication vs. Over cut off* | 28 | 3.9 | 2.2-7.2 | 16 | 5.5 | 2.2-13.6 | 8 | 3.3 | 1.1-9.7 |
|  | **Frequency of alcohol intoxication during last 30 days**  *0 times vs. ≥3 times* | 23 | 2.9 | 1.7-4.8 | 12 | 3.5 | 1.7-7.2 | 10 | 4.8 | 2.1-11.2 |
| 2. | **Age of first intoxication**  *No intoxication vs.≤12 yrs.* | 24 | 5.0 | 2.6-9.7 | 13 | 7.6 | 2.9-20.1 | 6 | 2.6 | 0.8-8.9 |
|  | **Number of drinks needed to feel intoxicated**  *No intoxication vs. Over cut off* | 27 | 2.9 | 1.6-5.6 | 15 | 4.7 | 1.8-12.2 | 8 | 2.0 | 0.6-6.3 |
|  | **Frequency of alcohol intoxication during last 30 days**  *0 times vs. ≥3 times* | 22 | 2.1 | 1.2-3.7 | 11 | 2.8 | 1.2-6.2 | 10 | 3.2 | 1.3-8.0 |
| 3. | **Age of first intoxication**  *No intoxication vs.≤12 yrs.* | 21 | 4.5 | 2.2-9.2 | 10 | 5.2 | 1.9-14.7 | 4 | 1.6 | 0.4-6.2 |
|  | **Number of drinks needed to feel intoxicated**  *No intoxication vs. Over cut off* | 26 | 3.1 | 1.6-6.0 | 14 | 4.4 | 1.7-11.5 | 7 | 1.7 | 0.5-5.6 |
|  | **Frequency of alcohol intoxication during last 30 days**  *0 times vs. ≥3 times* | 19 | 1.9 | 1.0-3.4 | 8 | 2.2 | 0.9-5.3 | 8 | 2.5 | 0.9-6.6 |

^1^Youth Self Report
